# Clinical Metabolomics Identifies Blood Serum Branched Chain Amino Acids as Potential Predictive Biomarkers for Chronic Graft vs. Host Disease

**DOI:** 10.3389/fonc.2019.00141

**Published:** 2019-03-18

**Authors:** Marcos Rodrigo Alborghetti, Maria Elvira Pizzigatti Correa, Jennifer Whangbo, Xu Shi, Juliana Aparecida Aricetti, Andreia Aparecida da Silva, Eliana Cristina Martins Miranda, Mauricio Luis Sforca, Camila Caldana, Robert E. Gerszten, Jerome Ritz, Ana Carolina de Mattos Zeri

**Affiliations:** ^1^Department of Cell Biology, University of Brasilia, Brasilia, Brazil; ^2^Hematology and Hemotherapy Center, Instituto Nacional de Ciência e Tecnologia do Sangue, University of Campinas, Hemocentro-Unicamp, Campinas, Brazil; ^3^Harvard Medical School, Dana-Farber Cancer Institute, Boston, MA, United States; ^4^Beth Israel Deaconess Hospital, Harvard Medical School, Boston, MA, United States; ^5^Brazilian Bioethanol Science and Technology Laboratory (CTBE)/Brazilian Center for Research in Energy and Materials (CNPEM), Campinas, Brazil; ^6^Brazilian Biosciences National Laboratory (LNBio), Brazilian Center for Research in Energy and Materials (CNPEM), Campinas, Brazil

**Keywords:** graft vs. host disease, bone marrow transplantation, cancer, metabolomics, branched chain amino acids, biomarkers, mass spectrometry, nuclear magnetic resonance

## Abstract

The allogeneic hematopoietic stem cell transplantation procedure—the only curative therapy for many types of hematological cancers—is increasing, and graft vs. host disease (GVHD) is the main cause of morbidity and mortality after transplantation. Currently, GVHD diagnosis is clinically performed. Whereas, biomarker panels have been developed for acute GVHD (aGVHD), there is a lack of information about the chronic form (cGVHD). Using nuclear magnetic resonance (NMR) and gas chromatography coupled to time-of-flight (GC-TOF) mass spectrometry, this study prospectively evaluated the serum metabolome of 18 Brazilian patients who had undergone allogeneic hematopoietic stem cell transplantation (HSCT). We identified and quantified 63 metabolites and performed the metabolomic profile on day −10, day 0, day +10 and day +100, in reference to day of transplantation. Patients did not present aGVHD or cGVHD clinical symptoms at sampling times. From 18 patients analyzed, 6 developed cGVHD. The branched-chain amino acids (BCAAs) leucine and isoleucine were reduced and the sulfur-containing metabolite (cystine) was increased at day +10 and day +100. The area under receiver operating characteristics (ROC) curves was higher than 0.79. BCAA findings were validated by liquid chromatography coupled to tandem mass spectrometry (LC-MS/MS) in 49 North American patients at day +100; however, cystine findings were not statistically significant in this patient set. Our results highlight the importance of multi-temporal and multivariate biomarker panels for predicting and understanding cGVHD.

## Introduction

Allogeneic hematopoietic stem cell transplantation (HSCT) is the only curative therapy available for several hematological malignances (1). Despite the improvements in survival following allogeneic HSCT, chronic graft vs. host disease (cGVHD) remains the most important cause of morbidity and late mortality for long-term transplant survivors (2–5). cGVHD is a multisystem disorder with alloreactive T cells (5), dendritic cells (DC) (6) and donor B cells (7) implicated in the pathogenesis. Although risk factors are associated with cGVHD incidence (patient age, MHC compatibility, female donor to male patient), the diagnosis relies almost entirely on the presence of clinical symptoms, generally in a stage with high grade inflammatory infiltrates and target organ damage (8, 9). Currently, no laboratory or molecular tests exist for cGVHD prediction prior to symptom development (10). For cGVHD, few groups have reported potential biomarkers and a panel has not been developed as for aGVHD (11). In 2007, high levels of B-Cell Activation Factor were observed in patients with active cGVHD (12), and in 2014 the protein CXCL9, a T-cell chemoattractant, was identified as a potential biomarker for the onset of newly diagnosed cGVHD in patients (2). Both protein concentrations were measured in plasma samples through hypothesis-driven projects for cGVHD diagnosis. Other inflammatory and regulatory proteins may also be associated with an increased risk for cGVHD, such as higher levels of TNFa and IL-10 (13, 14) and decreased TGFb (14, 15) and IL-15 (16).

Impressive progress has been made in “Omics” sciences; metabolomics analyses—complementary to genomic, transcriptomic, and proteomic—are capable of producing a more detailed and systematic picture of cellular processes and their response to changes in the environment. This dynamic nature is not always represented by only one biomarker, but by biomarker signatures, that increase the sensitivity and specificity required by clinical diagnosis (17). Toward this end, the NMR (nuclear magnetic resonance)- and mass-spectrometry-based metabolomics approaches enable the detection and absolute quantification of dozens of metabolites (e.g., amino acids, carbohydrates, fatty acids, keto acids) simultaneously, in a hypothesis-free approach, a very important feature for biomarker discovery studies (18). The interaction between genetic factors (e.g., mutations, epigenetic, single nucleotide polymorphisms) and lifestyle factors (age, diet, exercise, drugs, hormonal homeostasis, health-to-disease status, gut microbiota) will affect the pool of metabolites in a given biofluid at a given timepoint (19–21), allowing the use of metabolomic data for multivariate analysis and modeling in pathology studies.

However, to date, only a few reports have been published exploring metabolomics in studies involving HSCT. One study in animal models implicates glutathione dysregulation in an experimental GVHD model and branched chain amino acids (BCAA) with hepatic injury (22). Another study analyzed pretransplant the metabolic profiles of patients who had undergone HSCT implicated BCAA, among other metabolites, to the risk of developing acute GVHD (23). In the present work, multi-platform metabolomics facilities profiled blood serum prospectively collected (day −10 to day +100) in a Brazilian cohort, creating a panel with potential biomarkers predictive for cGVHD. This panel was further validated in plasma from a North American cohort (day +100). Our results demonstrated that BCAAs are potential prediction biomarkers for cGVHD in both cohorts, and sulfur-containing metabolites (precursors of GSH) could also be implicated in the pathology.

## Patients and Methods

**Figure 1:**
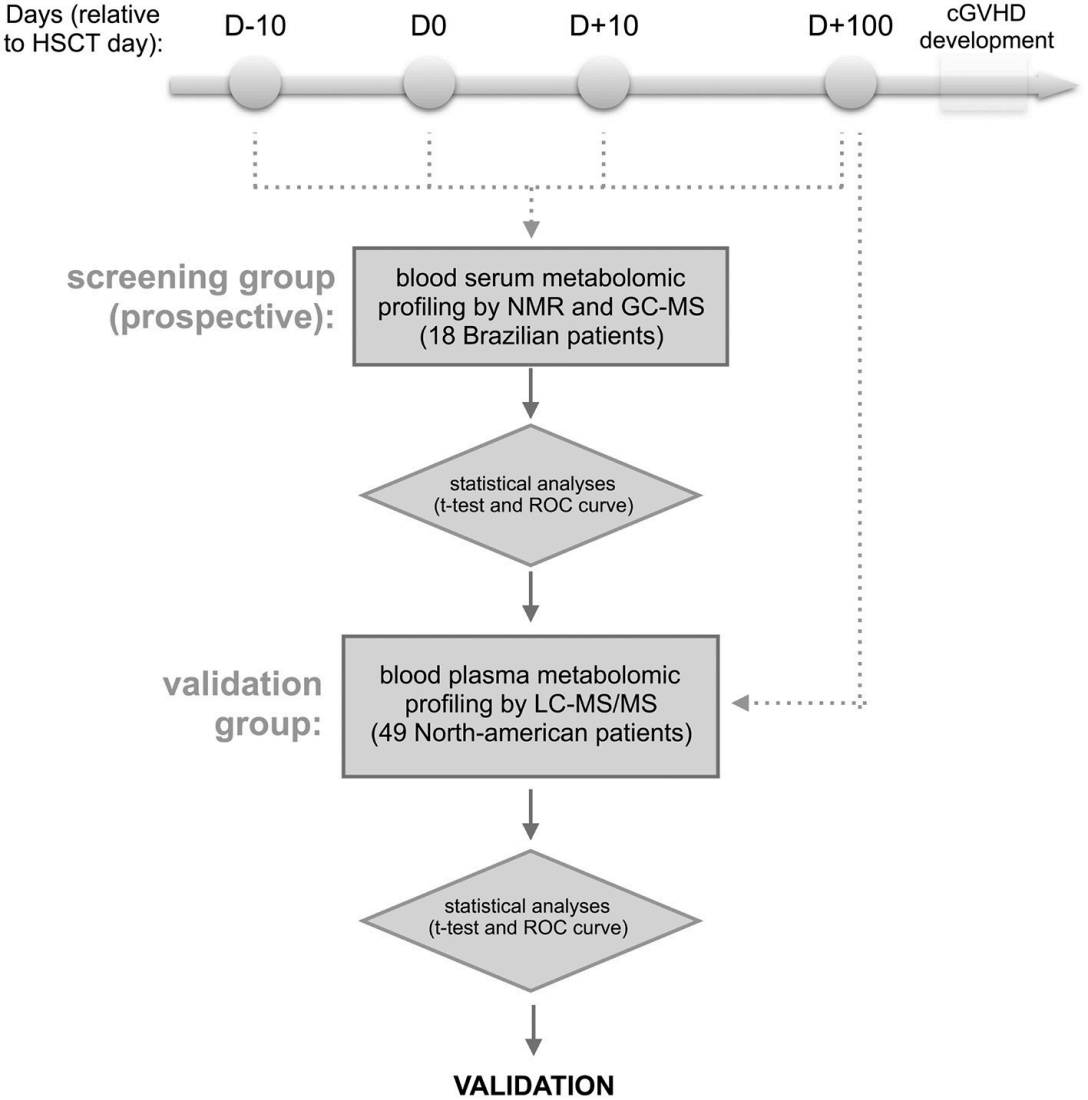
Experimental design. The approach involves the targeted serum metabolic profiling (by NMR and GC-MS) of Brazilian patients undergoing allogeneic hematopoietic stem cell transplantation (discovery cohort). Blood serum samples were prospectively collected the day before starting the myeloablative conditioning regimen (D-10), HSCT day (D0), as well as 10 and 100 days after HSCT (D+10 and D+100, respectively). The retro-prospective statistical analysis (uni- and multivariate) aimed to create a panel of potential biomarkers for cGVHD. This panel was further validated in North American patients' plasma collected at day +100 by targeted metabolic profiling LC-MS/MS. Any patients showed aGVHD or cGVHD symptoms before or at sampling day.

The outline of the methodology is shown in Figure 1. Briefly, blood samples were collected from patients who were recipients first of HSCT, and who did not develop aGVHD or present clinical symptoms of cGVHD at collection time. The blood serum samples from the discovery cohort were collected in four different periods of HSCT (at day −10, 0, +10, and +100) from 18 Brazilian patients, and the samples from day +100 of validation cohort were obtained from 49 North American patients. For discovery cohort, metabolites were identified and absolute quantification was determined by nuclear magnetic resonance (NMR) (24, 25) and the measurements were also verified by gas chromatography coupled to mass spectrometry (GC-MS) (26–28). After follow-up, patients were divided into 2 groups—patients that developed cGVHD and patients that did not develop cGVHD (cGVHD-free) (29)—and uni- and multivariate statistical analyses were performed. Results from the discovery cohort were validated by relative quantification of metabolites by liquid-chromatography coupled to tandem mass spectrometry (LC-MS/MS) (30, 31) in the validation cohort.

### Patients and Sample Collecting

This study included a prospective, nested cohort of 41 adult Brazilian patients who had undergone first full-match (related donor) allogeneic HSCT at the BMT Unit of the Clinical Hospital/University of Campinas between August 2011 and January 2013. All patients signed the Consent Form approved by the School of Medicine Institutional Review Board (n.1062/2010) of the University of Campinas, Brazil. Serum samples were prospectively collected in four different periods of the HSCT: on the day of hospitalization, D-10 (average day −7, min −12, max −5), on the day of transplantation, D0 (average day 0, min 0, max 0), during the neutropenic period, D+10 (average day 10, min 7, max 14), and D+100 (average day 99, min 75, max 126) post-HSCT (Figure 1). Blood samples were collected in serum clot activator with gel separator tubes after an overnight fast. These samples were centrifuged 10 min, 3,000 rpm at room temperature, aliquoted and immediately stored at −80°C until analyses by nuclear magnetic resonance (NMR) and gas chromatography coupled with time-of-flight (GC-TOF) mass spectrometry.

Findings from the Brazilian cohort were examined in a validation cohort of 49 North Americans who had undergone allogeneic HSCT between January 2004 and January 2013. Blood plasma samples from D+100 (average day 98, min 81, max 120) post-HSCT were selected from a sample bank of HSCT patients at the Dana-Farber Cancer Institute (Boston, MA). At the time of blood collection, none of the patients had clinical symptoms of cGVHD or had developed aGVHD. After the blood collection, 25 patients developed cGVHD and 24 were cGVHD-free. Written informed consent was obtained from each patient and donor prior to sample collection, in accordance with the Declaration of Helsinki. Protocol approval was obtained from the Human Subjects Protection Committee of the Dana-Farber/Harvard Cancer Center. Samples from the validation cohort were prepared and analyzed by liquid chromatography coupled to tandem mass spectrometry (LC-MS/MS) (Figure 1).

### Metabolite Profiling Analyses by Nuclear Magnetic Resonance (NMR)

Samples from the discovery cohort were defrosted on ice. In order to remove proteins and insoluble impurities, samples were filtered in a 3 kDa cut-off filter (Millipore Microcron YM3) rinsed 4 times with water prior to use to remove the glycerol content from the membrane. The filtration was performed by centrifugation at 11,000 rpm, 4°C for 1 h. The filtered samples were diluted 3x in a final concentration of 100 mM phosphate buffer, with pH 7.4, 10% D_2_O and referenced with 0.5 mM DSS (4,4-dimethyl-4-silapentane-1-sulfonic acid) for further absolute quantification of metabolites (24). The samples (550 μL) were transferred to 5 mm NMR tubes (Norell® Standard Series™) for analysis.

The acquisition of spectra was performed on an Agilent/Varian INOVA600 spectrometer operating at ^1^H Larmor frequency of 599.887 MHz and temperature of 298 K. The spectrometer was equipped with a triple resonance cryogenic probe and a Z pulse-field gradient unit. 1D pre-saturation pulse sequence was collected with 256 transients and 4 steady-state scans using a 4 s acquisition time and a 1.5 s recycle delay.

Before the processing and spectra analyses, all FIDs were zero-filled to 64 k data points and subjected to line broadening of 0.5 Hz. The internal standard for chemical shift referencing (set to 0 ppm) and quantification was DSS's singlet. The spectra processing, identification and quantification of metabolites were performed by using the application package Chenomx NMR Suite and the target profiling approach (Chenomx Inc.), as previously described (25). All identified and quantified metabolite concentration information was exported from the spectra to an excel sheet. All metabolite concentrations, except for DSS, were multiplied by 3 in order to adjust for the dilution effect.

### Metabolite Profiling Analyses by Gas Chromatography Coupled to Time-of-Flight (GC-TOF) mass spectrometry

For metabolite extraction, 100 μL of serum from the discovery cohort was mixed with 1 mL of a precooled (−15°C) mixture of MTBE: methanol:water 3:1:1 (v/v/v), shaken for 30 min at 4°C and ultrasonicated for 10 min in an ice-cooled bath, as described previously. Three phases were obtained after the addition of methanol:water (3:1) and centrifugation for 5 min at 4°C (26). The 250 μl of the aqueous phase (middle phase) was dried and derivatized as described by Roessner et al. (27). One microliter of the derivatized samples were analyzed on a Combi-PAL autosampler (Agilent Technologies GmbH, Waldbronn, Germany) coupled to an Agilent 7890 gas chromatograph coupled to a Leco Pegasus 2 time-of-flight mass spectrometer (LECO, St. Joseph, MI, USA) as described by Weckwerth (32). QC (mix of 20 standards) and blank samples were included in the running queue after every 10 samples, and data was normalized by total ion counting. Additionally, internal standard (C13 sorbitol) was spiked during sample extraction to control extraction performance and for estimation of metabolite recovery. Chromatograms were exported from Leco ChromaTOF software (version 3.25) to R software. Peak detection, retention time alignment, and library matching were performed using Target Search R-package (28). Metabolites were quantified by peak intensity of a selective mass. Metabolite intensities were normalized by dividing by the sum of the total ion count.

### Polar Metabolite Profiling Analyses by Liquid Chromatography Coupled to Tandem Mass Spectrometry (LC-MS/MS)

Plasma metabolic profiling from the validation cohort was performed as described previously (30, 31). Briefly, polar metabolites were extracted from 10 μL of blood plasma via protein precipitation with nine additions of nine 74.9:24.9:0.2 vol/vol/vol acetonitrile/methanol/formic acid containing two additional stable isotope-labeled internal standards for valine-d8 and phenylalanine-d8. Samples were centrifuged (10 min, 15,000 *g*, 4°C) and the supernatants were injected directly. Liquid chromatography-tandem mass spectrometry data were acquired in a 4000 QTRAP triple quadrupole mass spectrometer (Applied Biosystem/Sciex). Hydrophilic interaction chromatography (HILIC, 150 × 2.1 mm Atlantis HILIC columns, Waters) was performed. MS analyses were carried out using electrospray ionization (ESI) and multiple reaction monitoring (MRM) scans in the positive ion mode. MultiQuant software (Version 1.1; Applied Biosystems/Sciex) was used for automated peak integration and metabolite peaks were manually reviewed for quality of integration.

### Cystine and Cysteine Measurements by Liquid Chromatography Coupled to Tandem Mass Spectrometry (LC-MS/MS)

For cystine and cysteine measurements in the validation cohort blood plasma deproteination was performed by ultrafiltration in a 3 kDa cut-off filter (Millipore Microcron YM3) previously rinsed 4 times with water to remove the glycerol content from the membrane. Ten microliters of filtered plasma was diluted in nine volumes of solution A (1:1 water + 0.1% formic acid: acetonitrile + 0.1% formic acid, 55.55 μM cystine-d4, 55.55 μM cysteine-d2, 0.21 μg/mL phenylalanine-d8) and injected directly. Liquid chromatography-tandem mass spectrometry (LC-MS) data were acquired using an Agilent 6490 Triple Quadrupole LC/MS/MS coupled to 1290 Infinity UHPLC. The two pumps were similarly configured for hydrophillic interaction chromatography (HILIC) using 150 × 2.1 mm Atlantis HILIC columns (Waters) and with the same mobile phases (mobile phase A: 10 mM ammonium formate and 0.1% formic acid, v/v; mobile phase B: acetonitrile with 0.1% formic acid, v/v). Each column was eluted isocratically with 5% mobile phase A for 1 min followed by a linear gradient to 60% mobile phase A over 10 min. MS analyses were carried out using electrospray ionization (ESI) and multiple reaction monitoring (MRM) scans in the positive ion mode. Declustering potentials and collision energies were optimized for each metabolite by infusion of reference standards prior to sample analyses. The dwell time for each transition was 30 ms, the ion spray voltage was 4.5 kV, and the source temperature was 425°C. Internal standard peak areas were monitored for quality control and individual samples with peak areas differing from the group mean by more than two standard deviations were re-analyzed. Analyst software was used for automated peak integration and metabolite peaks were manually reviewed for quality of integration and compared against a known standard to confirm identity.

### Statistical Analyses

For the screening cohort (Brazilian patients), the statistical study included patients whose blood samples were collected throughout all periods of the study (day −10, day 0, day +10 and day +100, relative to HSCT day). Patients who had at least one missing sample, patients who died for reasons other than cGVHD and patients who developed aGVHD anytime or cGVHD at any sampling time, were excluded from the statistical analysis. The statistical analyses aimed to compare the metabolic differences between patients who developed cGVHD after blood collection and patients with no cGVHD diagnoses (cGVHD-free) during follow up. In order to obtain significant predictors for cGVHD, cases and controls were compared by univariate and multivariate analysis. The independent variable was set as cGVHD-free or cGVHD group (29). Only data with normal distributions (Shapiro-Wilk normality test) were analyzed by *t*-test with no further normalization. Student's *t*-test followed by hierarchical clustering method and Heatmap visualization were applied. For Heatmap visualization, data were autoscaled. The distance measurement was Euclidean and the clustering algorithm was Ward. The best clustering result, for cGVHD-free vs. cGVHD groups, was selected with the minimum metabolites possible. Area under curve (AUC) from univariate and multivariate receiver operating characteristic (ROC) analyses and corresponding 95% confidence intervals were calculated to estimate the clinical potential of the selected metabolites as prognostic cGVHD biomarkers (18). The statistical analyses were performed using the software Prism 6.0f (GraphPad Software, Inc.) and the web tools MetaboAnalyst 2.0 and ROCCET (18, 33). The validation of results was performed in 49 North-American patients (24 cGVHD-free and 25 cGVHD) with plasma samples collected around day +100 by univariate analysis (student *t*-test) and ROC curve analyses. Statistical analyses correlating data from different platforms (NRM, GC-MS and LC-MS/MS) were not performed.

## Results

We hypothesized that changes in the metabolic profile of blood precede cGVHD's clinical symptoms. Using a discovery cohort (Brazilians), we performed metabolomic profiling on serum blood collected on days −10, 0, +10, and +100, referred by HSCT day (Figure 1). From 41 adult patients who had undergone first fully-matched related donor allogeneic HSCT between August 2011 and January 2013, 18 (44%) patients were enrolled on this study. None of these patients presented with cGVHD or aGVHD symptoms at or before the day of collection. Patient median age was 36 years (21–69 years), 8 patients (44%) were female and 5 (28%) were male receptors from female donors. Six (33%) patients developed cGVHD and 12 (67%) patients were cGVHD-free.(29) The median time of cGVHD diagnosis was 179 days (124–246 days) and the follow-up median was 459 days (156–679 days). Characteristics of the study population are shown on Table 1. Sixty-three (63) metabolites were identified and quantified in serum blood samples of these patients by the NMR-based metabolomic platform (Supplementary Table 1).

**Table 1:** Demographic and characteristics of discovery cohort (Brazilians).

**Characteristics**	**Total (*n* = 18)**	**cGVHD-Free (*n* = 12)**	**cGVHD (*n* = 6)**
**Patient age, median (range), y**	36 (21–69)	35 (21–61)	47 (34–69)
**Patient gender, no. (%)**			
Female	8 (44)	6 (50)	2 (33)
**Diagnosis at transplant, no. (%)**			
Acute myeloid leukemia	9 (50)	8 (67)	1 (17)
Chronic myeloid leukemia	1 (6)	1 (8)	0 (0)
Non-malignant Disorders	2 (11)	0 (0)	2 (33)
Others	6 (34)	3 (25)	3 (50)
Donor age, median (range), y	38 (19–69)	38 (26–58)	47 (19–69)
**Receptor/Donor gender, no. (%)**			
Male/female	5 (28)	3 (25)	2 (33)
Others	13 (72)	8 (67)	4 (67)
**Donor type, no. (%)**			
HLA-identical related	18 (100)	12 (100)	6 (100)
**Conditioning regimen types, no. of Patients (%)**			
Busulfan and Cyclophosphamide	11 (61)	8 (67)	3 (50)
Busulfan and Fludarabine	2 (11)	1 (8)	1 (17)
Fludarabine and TBI	2 (11)	0 (0)	2 (33)
Other	3 (17)	3 (25)	0 (0)
**Source of stem cells, no. of Patients (%)**			
Bone marrow	8 (44)	6 (50)	2 (33)
Peripheral blood	10 (56)	6 (50)	4 (67)
**GVHD prophylaxis, no. of Patients (%)**			
Cyclosporine+ Methotrexate	16 (89)	12 (100)	4 (67)
Cyclosporine+ Mycophenolate mofetil	2 (11)	0 (0)	2 (33)

**Figure 2:**
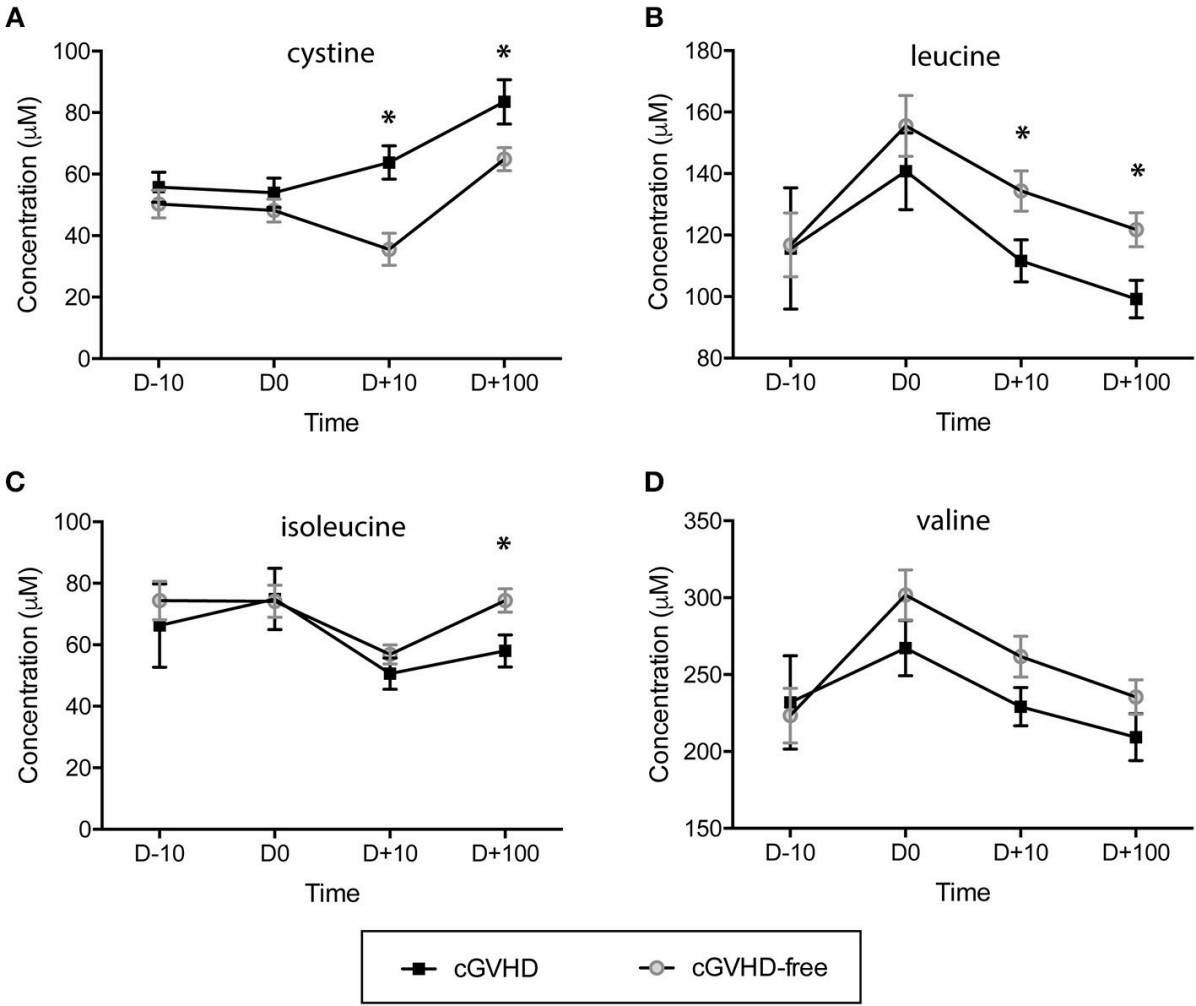
Branched chain amino acids (BCAAs) are reduced and the sulfur-containing metabolite cystine is increased in patients prone to developing cGVHD. Concentration dynamics of potential predictive cGVHD biomarkers along allogeneic HSCT in the discovery group. From 63 metabolites measured by NMR at blood serum, 3 displayed concentration patterns important for differentiating cGVHD patients from patients without cGVHD by hierarchical clustering analyses (HCA). Valine concentration is included because it is a branched chain amino acid (as leucine and isoleucine). The day before starting the myeloablative conditioning regimen is represented by D-10, D0 is the HSCT day, D+10 and D +100 are 10 and 100 days after HSCT. **(A)** Cystine, **(B)** Leucine, **(C)** Isoleucine, **(D)** Valine. Bars: standard error mean. Asterisk: student's *t*-test *p* < 0.05.

The concentration of sulfur-containing metabolite cystine was found higher in cGVHD patients at day +10 (63.8 ± 5.4 μM) and day +100 (83.5 ± 7.2 μM) when compared to cGVHD-free patients at day +10 (35.6 ± 5.2 μM) and day +100 (64.9 ± 3.7 μM), with student *t*-test *p* = 0.004 (day +10) and *p* = 0.021 (day +100) (Figure 2A). In contrast to cystine, branched chain amino acids displayed reduced concentrations in the cGVHD group when compared to the cGVHD-free group (Figures 2B–D). Leucine concentrations (Figure 2B) were lower in patients that developed cGVHD at day +10 (111.6 ± 6.8 μM vs. 134.4 ± 6.5 μM cGVHD vs. cGVHD free patients, *p* = 0.045) and day +100 (99.2 ± 6.1 μM vs. 121.8 ± 5.5 μM, *p* = 0.023). Isoleucine concentrations (Figure 2C) were lower in cGVHD patients at day +100 (58.0 ± 5.2 μM) than in cGVHD-free patients (74.4 ± 3.8 μM), *p* = 0.023. The concentration of valine (Figure 2D) also tended to be lower in cGVHD patients. BCAA and cystine concentrations were verified by GC-MS (Supplementary Figure 1, in arbitrary units) and were in good agreement with NMR measurements, except for cystine.

**Figure 3:**
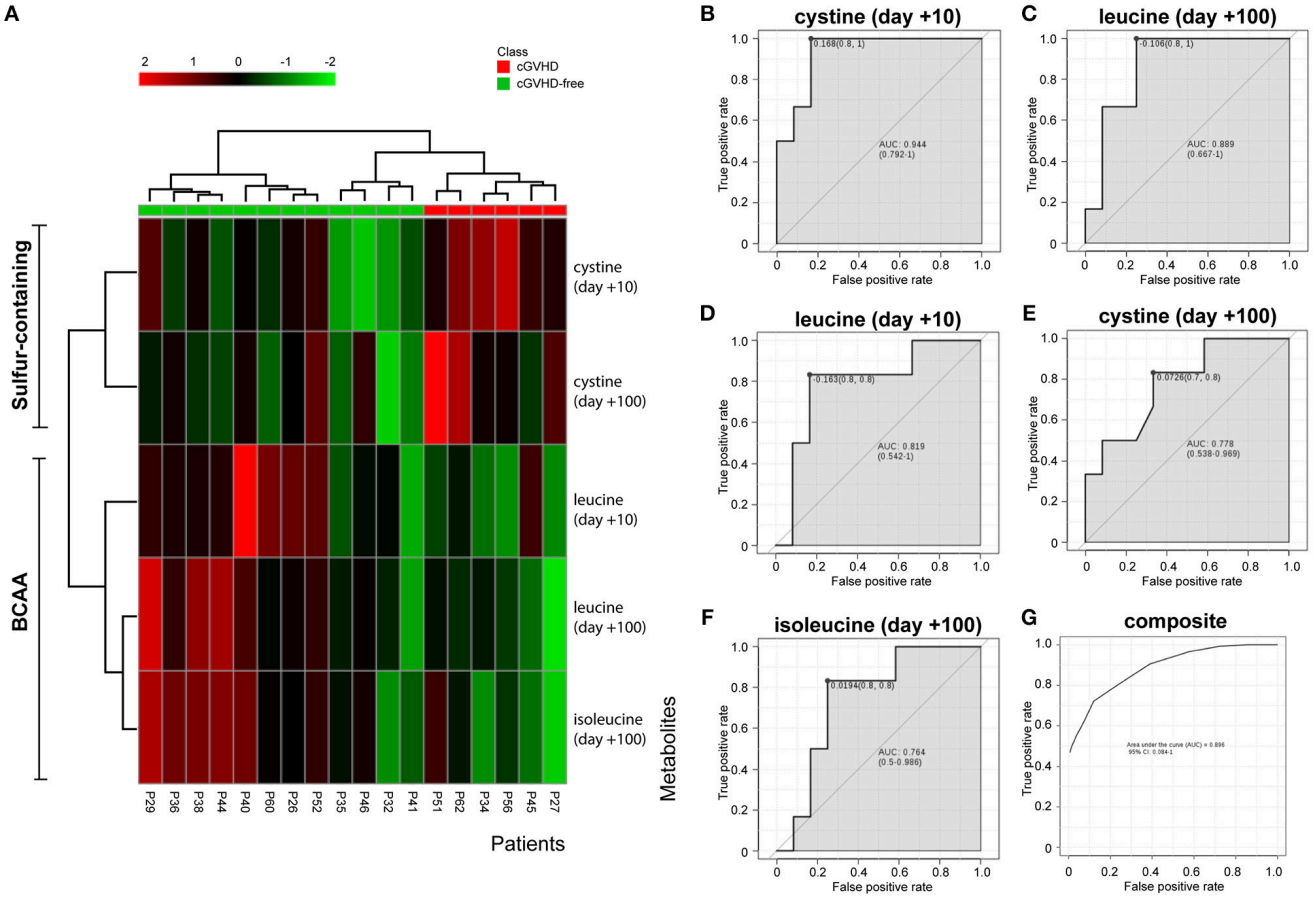
BCAA and cystine cluster cGVHD patients from patients without cGVHD. Clustering analyses and biomarker potential of 3 metabolites in 2 data points in the discovery cohort. **(A)** Heatmap generated by cystine and leucine auto-scaled concentrations at day +10 and day +100 and isoleucine at day +100. **(B–F)** Individual receiver operating characteristic (ROC) curves of metabolites and **(G)** composite ROC curve of the metabolite panel.

The variables identified by the *t*-test (cystine day +10, cystine day +100, leucine day +10, leucine day +100 and isoleucine day +100) were subject to hierarchical clustering analysis to generate a heatmap in order to investigate group segregation. As shown in Figure 3A, these variables were able to cluster cGVHD patients from cGVHD-free patients. Principal component analyses (PCA, Supplementary Figure 2A) displayed different segregation patterns for cGVHD patients and cGVHD-free patients by using 2 principal components (PC). PC1 explained 47.1% of variance and PC2 explained 29.3%. Partial least squares Discriminant Analysis (PLS-DA, Supplementary Figure 2B) also discriminates both groups (PC1 41.7% and PC2 34.4%, accuracy 0.889, R2 0.81 and Q2 0.69, 2000 permutation test *p* = 0.022).

ROC curve analyses were performed to better assess the clinical utility of each metabolite (as individual or composite) for cGVHD prediction (Figures 3B–G). All area under curve (AUC) measurements generated were higher than 0.76. Cystine at day +10 resulted in the best curve (Figure 3B, AUC 0.944, CI 0.792–1, *p* = 0.004), followed by leucine at day + 100 (Figure 3C, AUC 0.889, CI 0.667–1, *p* = 0.011), leucine at day +10 (Figure 3D, AUC 0.819, CI 0.542–1, *p* = 0.049), cystine at day +100 (Figure 3E, AUC 0.778, CI 0.548–0.969, *p* = 0.055) and isoleucine at day +100 (Figure 3F, AUC 0.764, CI 0.5–0.986, *p* = 0.031). The composite panel ROC curve AUC was 0.896 (Figure 3G, CI 0.084–1), better than all other metabolites but cystine at day +10.

We were not able to detect and quantify cysteine (the reduced form of cystine) in our NMR platform analyzing the Brazilian cohort samples. However, our GC-MS platform detected this metabolite, whose concentrations were not statistically different between the groups at any time (Supplementary Figures 3A,B, *p* > 0.5 *t*-test) but numerically higher in cGVHD patients. Two metabolites from BCAA metabolism were detected and quantified by our NMR platform: 2-oxoisocaproate (from leucine) and 3-methyl-2-oxovalerate (from isoleucine). Both products were reduced in cGVHD patients mainly at day +100 (Supplementary Figure 4). The 2-oxoisocaproate concentration mean was 6.7 μM (±0.8 μM) in patients without cGVHD and 4.9 μM (±0.2 μM) in cGVHD patients (Supplementary Figure 4A, *p* = 0.140). The 3-methyl-2-oxovalerate concentration mean was 5.8 μM (±0.7 μM) in patients without cGVHD and 3.9 μM (±0.3 μM) in cGVHD patients (Supplementary Figure 4B, *p* = 0.058).

**Figure 4:**
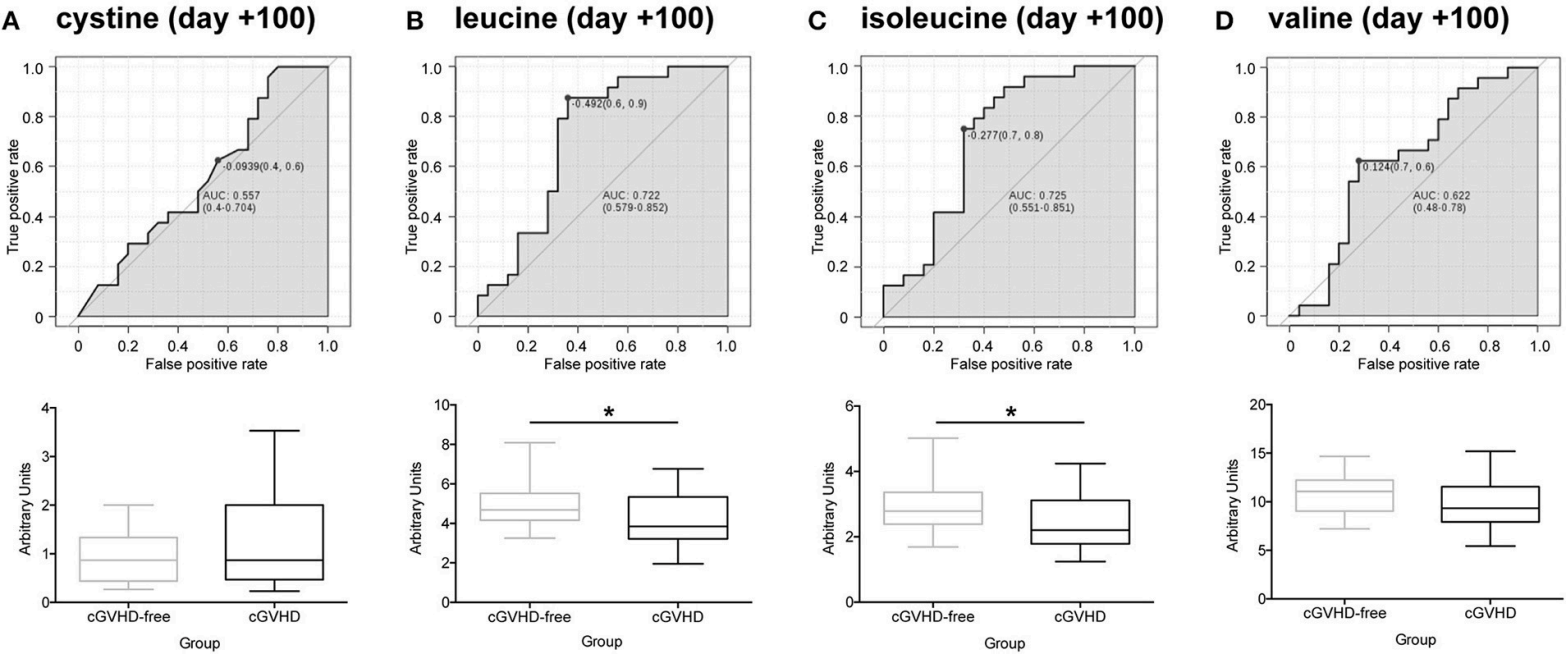
Validation of potential biomarkers in North American patients at day +100 by LC-MS/MS. ROC curves and box-plot of concentrations (in arbitrary units) are displayed. **(A)** Cystine, **(B)** Leucine, **(C)** Isoleucine, **(D)** Valine. Asterisk: student's *t*-test *p* < 0.05.

We obtained 49 plasma samples collected around day +100 (98 ± 10) from the Danna-Farber Cancer Institute to validate our findings. Again, none of the patients had clinical symptoms of cGVHD or aGVHD before the blood sampling. All patients had undergone allogeneic HSCT between January 2004 and January 2013. Patient median age was 54 years (25–73 years), 25 patients (51%) were female and 24 (49%) were male receptor female donors. Twenty-five (51%) selected patients developed cGVHD and 24 (49%) patients were cGVHD-free (29). Demographics and characteristics of the study population are shown in Supplementary Table 2. These samples were analyzed by LC/MS/MS in order to verify sulfur-containing metabolites (cystine and cysteine) and BCAA concentrations in a different cohort, from an outside transplantation center and from a diverse ethnic population. We were not able to replicate our findings for cystine in the North American cohort (Figure 4A). However, contrary to the findings in the Brazilian cohort (Supplementary Figure 3B), cysteine was found to be increased in cGVHD patients from the North American cohort (Supplementary Figure 3C, *p* = 0.007 *t*-test). Leucine (Figure 4B, *p* = 0.020) and isoleucine (Figure 4C, *p* = 0.028) significantly reduced in cGVHD patients by the *t*-test, while valine was numerically lower but did not reach statistical significance (Figure 4D, *p* = 0.160). ROC curve analysis of BCAA concentrations (excluding valine) from the validation cohort confirmed the ability of the score to predict cGVHD prior to symptom development. The area under curve for leucine was 0.721 (CI 0.577–0.862, *p* = 0.0083), for isoleucine it was 0.719 (CI 0.564–0.852, *p* = 0.015) and for valine it was 0.643 (CI 0.472–0.792, *p* = 0.119).

**Figure 5:**
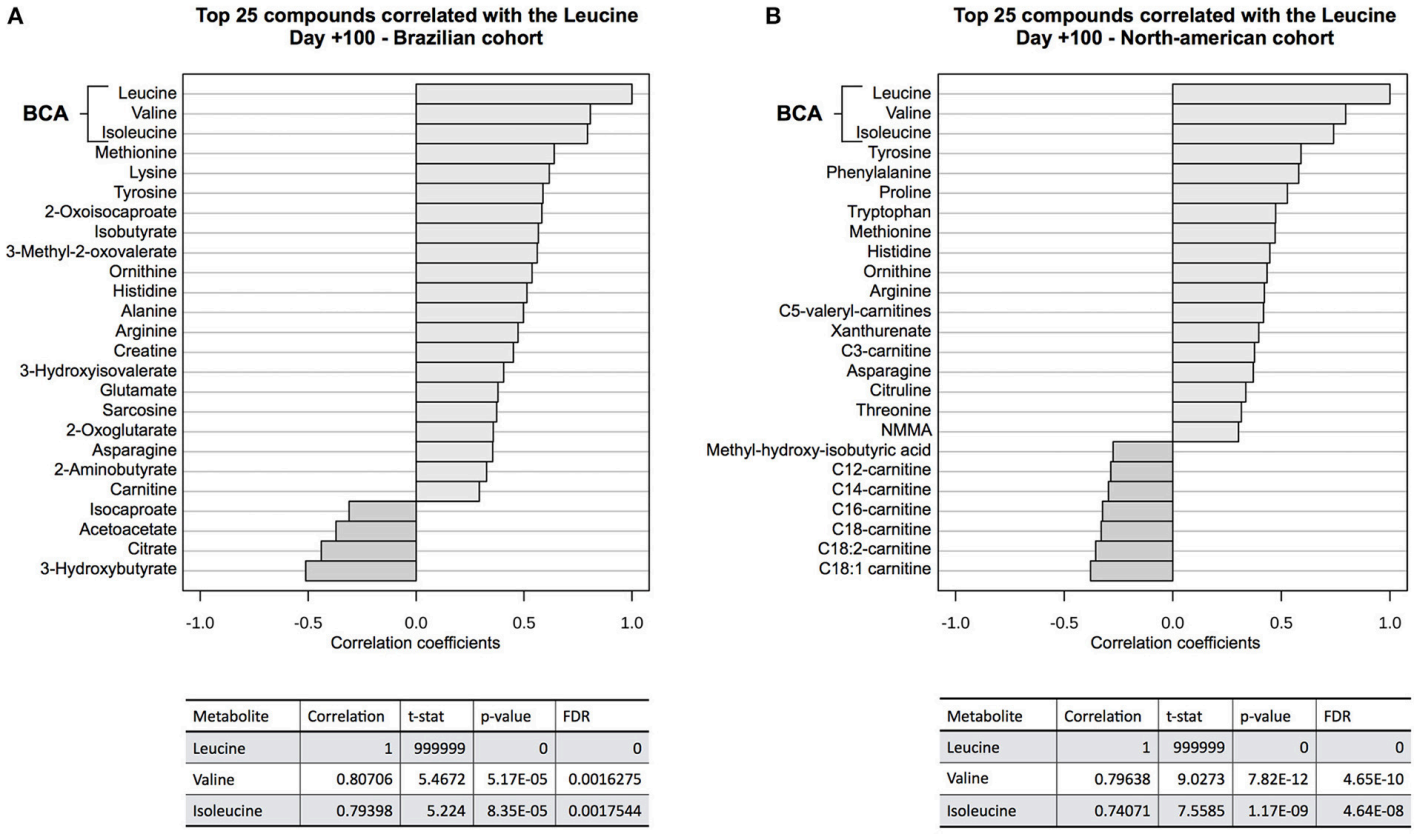
Correlation analysis to leucine at day +100. BCAA blood concentrations were well correlated to leucine concentration at **(A)** discovery cohort and **(B)** validation cohort. Other correlated metabolites in both cohorts were tyrosine, histidine, arginine, ornithine, methionine, and asparagine, showing evidence of overall consistence of metabolic profile measurements in both cohorts. 2-oxo-isocaproate and 3-methyl-2oxovalerate, BCAA degradation products, were also well-correlated in the discovery cohort. These BCAA degradation products were not detected by LC-MS/MS platform on validation cohort.

Correlation analyses (Figure 5) showed that valine and isoleucine concentrations are markedly correlated to leucine concentration in both cohorts, using two different metabolomics platforms (NMR and LC-MS/MS). The valine correlation coefficient was 0.807 (*p* = 5.2E-05) in the Brazilian cohort and 0.796 (*p* = 7.8E-12) in the North American cohort. The isoleucine correlation coefficient was 0.794 (*p* = 8.4E-05) and 0.741 (*p* = 1.2E-09) in Brazilians and North Americans, respectively. These results highlight the strong concentration correlation in BCAA and corroborate differences in both groups (cGVHD-free vs. cGVHD), indicating that if one BCAA changes, the others are concordantly changed. Moreover, BCAA oxidation products were also correlated to leucine in the Brazilian cohort: 0.582 (*p* = 0.011) and 0.561 (*p* = 0.015) for 2-oxoisocaproate and 3-methyl-2-oxovalerate, respectively. These oxidation products were not measured in the North American cohort.

## Discussion

Graft vs. host disease (GVHD) is the leading cause of morbidity and non-relapse mortality for long-term transplant survivors.(2–5, 34) Immunosuppressive therapy currently employed to treat cGVHD could be applied to prevent cGVHD(34), by delaying planned immunosuppression (2). However, no biomarker panel for cGVHD prediction is available, even with several approaches that could be applied for biomarker prospection. It is known that multisystem metabolism is altered in cGVHD. The metabolic state of B cells is heightened in patients with active cGVHD (35), as is the thiol/redox pathway in a GVHD experimental model (22). Because metabolism is highly dynamic, metabolite concentrations respond in a real-time fashion to reflect whole-body homeostasis. In this scenario, metabolomics emerges as a powerful approach with sensitivity and sensibility to detect these dynamic changes. The hypothesis of this study is that metabolomic profile changes in the blood precede the appearance of cGVHD clinical symptoms as tissues are damaged. In order to define when metabolomics changes would become evident, several blood collection times, and metabolic profiling (by NMR and GC-TOF-MS) were performed in the discovery cohort (Brazilians) at day −10, day 0, day +10 and day +100. An important aspect to highlight is that no patient had clinical symptoms of cGVHD or aGVHD at sampling time (the same is true for the validation cohort). Moreover, the validation cohort (North Americans) is from another transplant center, from a diverse ethnic population, and the metabolite profiling was performed by a distinct technique (LC/MS/MS), avoiding bias from population and analytic methodology, respectively. For the discovery cohort, sulfur-containing metabolite cystine and branched chain amino acids (BCAA, leucine, and isoleucine) were important to cluster cGVHD patients from patients without cGVHD before clinical symptoms at day +10 and day +100. We were able to replicate findings for BCAA in the validation group at day +100 but not cystine. However, cysteine, another sulfur-containing metabolite, was found increased in the North American cohort.

Cystine is an oxidized dimeric form of cysteine and is involved in T cell activation and proliferation. An excellent review on this process can be reviewed in the work by Yan (36). Shortly: naïve T cell activation and proliferation requires cysteine for glutathione (GSH) synthesis in their intracellular space, but these cells are unable to import cysteine, the less abundant amino acid in the blood stream (36, 37). T cell activation also depends on the immunological synapse with dendritic cells (DCs), and DCs are able to import cystine, which is more abundant in the blood stream than cysteine (36, 38). In the cytoplasm of DC, cystine is converted to cysteine that is delivered in the immunological synapse, producing a redox potential suitable for T cell proliferation. Furthermore, T cells synthetize GSH, and being activated can import this cysteine. Our data does not allow us to explain whether cystine/cysteine is increased as a response of immunological activity or whether this increase is related to some intrinsic feature, unleashed after allogeneic HSCT, from patients prone to developing cGVHD. In this regard, a study with animal models and metabolomics showed accumulation of plasma GSH precursors (cystathionine, total cysteine), and its catabolite (cysteinylglycine) is probably due to impaired hepatic GSH synthesis in early GVHD (22). The impaired GSH synthesis in the liver during GVHD could make GSH precursors (as cystine/cysteine) available for use by dendritic cells to activate T-cells.

Three branched chain amino acids (BCAAs)—leucine, isoleucine and valine—are among the nine essential proteinogenic amino acids for humans (39). We were able to identify and quantify these three amino acids in both cohorts. We found the three BCAA concentrations reduced in patients that developed cGVHD at day +10 and day +100 at discovery cohort and reduced at day +100 at validation cohort (with different ranges and statistic significances). Moreover, the degradation products of leucine and isoleucine (2-oxoisocaproate and 3-methyl-2-oxovalerate) were also found reduced in the cGVHD group in the discovery cohort at day +100. In animal models for GVHD, BCAA plasma levels were also reduced and strongly correlated with histopathological changes in the liver of GVHD mice (22). However, we cannot be sure that BCAAs are organ-specific biomarkers due to multi-organ involvement in both cohorts. BCAAs are essential to cells of the immune system. Uptake of BCAAs during activation or mitotic stimulation is increased in T cells as so in B cells (40, 41). BCAAs are also frequently associated with benefits to body weight regulation and muscle protein synthesis (42). These metabolites are considered anabolic signals via mammalian target of rampamycin complex 1 (mTORC1) activation and reduction of protein breakdown (42–44). These same mechanisms (protein synthesis and mTORC1 activation) can be enrolled for immune cell proliferation (41, 45). Our results do not allow us to conclude that BCAA reduction in patients pre-stage for cGVHD development is due to consumption by immune cells and further activation/proliferation, consumption by other tissues in order to recovery or avoid damage, or both, because BCAAs are important for immune cell activation and also for tissue maintenance.

In summary, we found two potential classes of predictive biomarkers for cGVHD by using unbiased large-scale metabolomic approaches in prospective and serial sampling of blood serum/plasma. We demonstrated that BCAAs and BCAA degradation product concentrations were reduced at day +10 and day +100 post-HSCT in patients prone to developing cGVHD. BCAAs were also reduced in a validation cohort at day +100. We also found cystine concentrations higher in patients prone to developing cGVHD at day +10 and day +100. Cystine was not confirmed at the validation cohort but another sulfur-containing metabolite, cysteine, was present. Strong points of this project are the use of multi-platform metabolomics approaches to prospect and confirm results in different ethnic populations, avoiding technical and population bias. Further experiments are necessary to access the clinical applicability of BCAA as predictive biomarkers for cGVHD and our results highlight multi-temporal and multivariate modeling as an approach to increase the accuracy of predictions. Furthermore, experimental models of GVHD and supplementation with BCAA can shed light in new potential cGVHD pre-emptive therapy.

## Author Contributions

MA, MC, JW, RG, JR, AZ, and CC conceived and designed the experiments. MA, MS, XS, and JA performed the experiments. MA, EM, and AZ analyzed the data. MA and MC wrote the first draft of the manuscript. MC, JW, CC, RG, JR, and AZ contributed to the writing of the manuscript. MA, AS, and JW enrolled patients.

### Conflict of Interest Statement

The authors declare that the research was conducted in the absence of any commercial or financial relationships that could be construed as a potential conflict of interest.
