# Supplementary material for: Clinical Metabolomics Identifies Blood Serum Branched Chain Amino Acids as Potential Predictive Biomarkers for Chronic Graft vs. Host Disease
Source: Front Oncol. 2019 Mar 18;9:141. doi: 10.3389/fonc.2019.00141 (PMC6436081; doi:10.3389/fonc.2019.00141)
Supplement: Supplementary file 2 [file Table_2.docx]

Supplementary material

**Clinical metabolomics identifies blood serum branched chain amino acids as potential predictive biomarkers for chronic graft-versus-host disease**

**Running title**

Potential metabolic biomarkers for cGVHD

Marcos Rodrigo Alborghetti,^1^ Maria Elvira Pizzigatti Correa,^2^ Jennifer Whangbo^3^, Xu Shi^4^, Juliana Aparecida Aricetti,^5^ Andreia Aparecida da Silva,^2^ Eliana Cristina Martins Miranda,^2^ Mauricio Luis Sforca^6^, Camila Caldana,^5^ Robert E. Gerszten^4^, Jerome Ritz^3^ and Ana Carolina de Mattos Zeri^6,*^

^1^Department of Cell Biology, University of Brasilia, Brasilia, Brazil

^2^Hematology and Hemotherapy Center, University of Campinas/Hemocentro-Unicamp, Instituto Nacional de Ciência e Tecnologia do Sangue, Campinas, São Paulo, Brazil

^3^Dana-Farber Cancer Institute, Harvard Medical School, Boston, Massachusetts, United States of America

^4^Beth Israel Deaconess Hospital, Harvard Medical School, Boston, Massachusetts, United States of America

^5^Brazilian Bioethanol Science and Technology Laboratory (CTBE) / Brazilian Center for Research in Energy and Materials (CNPEM), Campinas-SP, Brazil

^6^Brazilian Biosciences National Laboratory (LNBio) / Brazilian Center for Research in Energy and Materials (CNPEM), Campinas-SP, Brazil

Supplementary Table 2. Demographic and characteristics of validation cohort (North Americans)

| **Characteristics** | **Total (n=49)** | **cGVHD-Free (n=24)** | **cGVHD (n=25)** |
| --- | --- | --- | --- |
| **Patient age**, median (range), y | 54 (25-73) | 54 (25-73) | 54 (34-69) |
| **Patient gender**, no. (%) |  |  |  |
| Female | 25 (51) | 8 (33) | 17 (68) |
| **Diagnosis at transplant,** no. (%) |  |  |  |
| Acute myeloid leukemia | 20 (41) | 8 (33) | 12 (48) |
| Non Malignant Disorders | 1 (2) | 0 (0) | 1 (4) |
| Others | 28 (57) | 16 (67) | 12 (48) |
| **Donor type,** no. (%) |  |  |  |
| HLA-identical related | 44 (90) | 23 (96) | 21 (84) |
| **Conditioning regimen types,** no. of Patients (%) |  |  |  |
| Busulfan and Cyclophosphamide | 38 (78) | 22 (92) | 16 (64) |
| Busulfan and Fludarabine | 3 (6) | 1 (4) | 2 (8) |
| Cyclophosphamide and TBI | 6 (12) | 0 (0) | 6 (24) |
| Other | 2 (4) | 1 (4) | 1 (4) |
| **GVHD prophylaxis,** no. of Patients (%) |  |  |  |
| Cyclosporine+Methotrexate | 1 (2) | 1 (4) | 0 (0) |
| Cyclosporine+Mycophenolate mofetil | 2 (11) | 0 (0) | 2 (33) |
| Tacromilus+Methotrexate | 7 (14) | 3 (13) | 4 (16) |
| Tacromilus+Rapamycin+Methotrexate | 21 (43) | 13 (54) | 8 (32) |
| Other | 4 (8) | 0 (0) | 4 (16) |
