# Supplementary material for: Clinical Metabolomics Identifies Blood Serum Branched Chain Amino Acids as Potential Predictive Biomarkers for Chronic Graft vs. Host Disease
Source: Front Oncol. 2019 Mar 18;9:141. doi: 10.3389/fonc.2019.00141 (PMC6436081; doi:10.3389/fonc.2019.00141)
Supplement: Supplementary file 3 [file Data_Sheet_1.docx]

Supplementary material

**Clinical metabolomics identifies blood serum branched chain amino acids as potential predictive biomarkers for chronic graft-versus-host disease**

**Running title**

Potential metabolic biomarkers for cGVHD

Marcos Rodrigo Alborghetti,^1^ Maria Elvira Pizzigatti Correa,^2^ Jennifer Whangbo^3^, Xu Shi^4^, Juliana Aparecida Aricetti,^5^ Andreia Aparecida da Silva,^2^ Eliana Cristina Martins Miranda,^2^ Mauricio Luis Sforca^6^, Camila Caldana,^5^ Robert E. Gerszten^4^, Jerome Ritz^3^ and Ana Carolina de Mattos Zeri^6,*^

^1^Department of Cell Biology, University of Brasilia, Brasilia, Brazil

^2^Hematology and Hemotherapy Center, University of Campinas/Hemocentro-Unicamp, Instituto Nacional de Ciência e Tecnologia do Sangue, Campinas, São Paulo, Brazil

^3^Dana-Farber Cancer Institute, Harvard Medical School, Boston, Massachusetts, United States of America

^4^Beth Israel Deaconess Hospital, Harvard Medical School, Boston, Massachusetts, United States of America

^5^Brazilian Bioethanol Science and Technology Laboratory (CTBE) / Brazilian Center for Research in Energy and Materials (CNPEM), Campinas-SP, Brazil

^6^Brazilian Biosciences National Laboratory (LNBio) / Brazilian Center for Research in Energy and Materials (CNPEM), Campinas-SP, Brazil


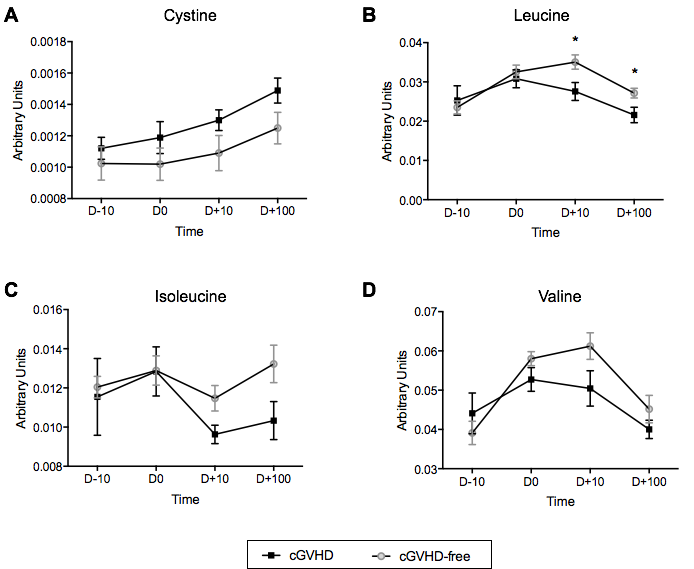


**Supplementary figure 1. Concentration dynamics (measured by GC-TOF-MS) of potential predictive cGVHD biomarkers along allogeneic HSCT in the discovery group.** The day before myeloablative conditioning regimen start is represented by D-10, D0 is the HSCT day, D+10 and D +100 are 10 and 100 days after HSCT. A) Cystine, B) Leucine, C) Isoleucine, D) Valine. Bars: standard error mean. Asterisk: student t test p value < 0.05. Isoleucine p value at day +100 was 0.07.


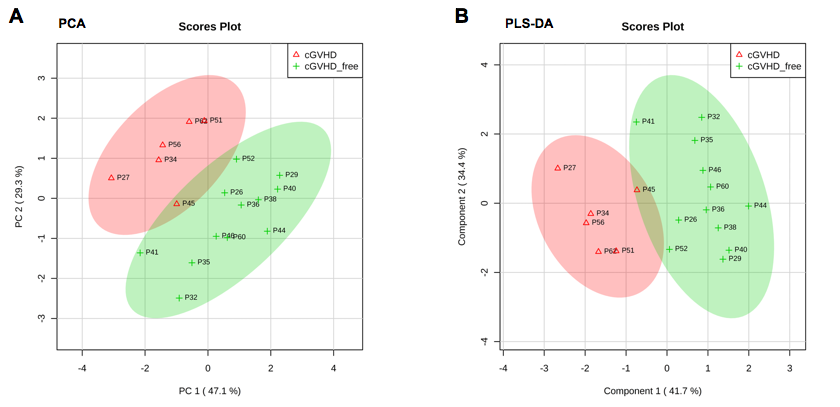


**Supplementary figure 2.** **Plasma BCAA and sulfur-containing metabolite cystine discriminate patients prone develop cGVHD and patients without cGVHD at day +10 and +100 in the discovery cohort.** A) Principal Component Analysis (PCA) scores plot and (B) Partial least squares Discriminant Analysis (PLS-DA) of patients prone to develop cGVHD (red) and without cGVHD (green) performed with leucine and cystine concentrations at day +10 and +100 and isoleucine at day +100. Cross validation (2 components): accuracy 0.889, R2 0.81, Q2 0.69); permutation: p = 0.022 (separation distance [BW], permutation numbers: 2000). The explained variances are shown in brackets.


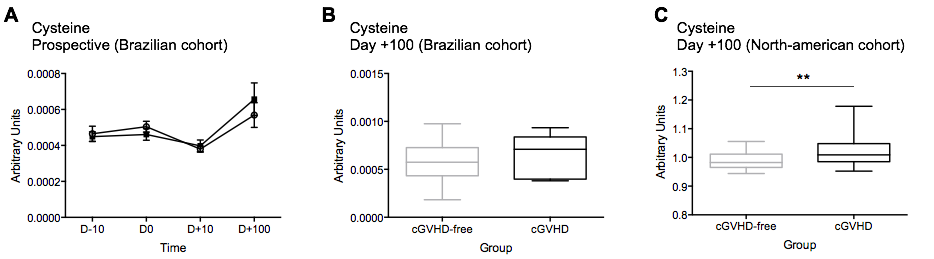


**Supplementary figure 3. Cysteine measurements.** A) Cysteine concentration dynamics (measured by GC-TOF-MS) along allogeneic HSCT in the discovery cohort. The day before myeloablative conditioning regimen start is represented by D-10, D0 is the HSCT day, D+10 and D +100 are 10 and 100 days after HSCT. Cysteine concentration box plots at (B) discovery cohort and (C) validation cohort at day +100. Bars: standard error mean. Double asterisk: student t test p value = 0.007.


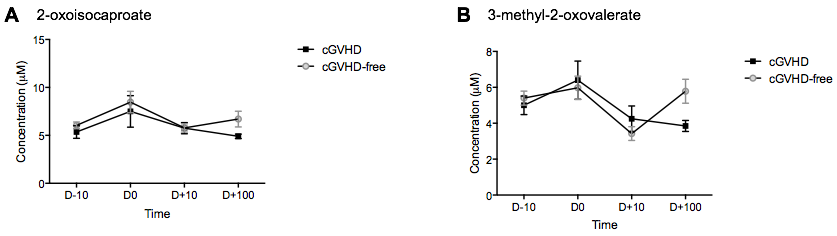


**Supplementary figure 4.** **BCAA degradation products measurements.** A) Leucine degradation product (2-oxoisocaproate) and (B) isoleucine degradation product (3-methyl-2-oxovalerate) concentration dynamics (measured by NMR) along allogeneic HSCT in the discovery cohort. The day before myeloablative conditioning regimen start is represented by D-10, D0 is the HSCT day, D+10 and D +100 are 10 and 100 days after HSCT. Cysteine concentration box plots at (B) discovery cohort and (C) validation cohort at day +100. Bars: standard error mean. Student t test p value =0.15 and = 0.06 for 2-oxoisocaproate and 3-methyl-2-oxovalerate at day +100, respectively.
